# Feasibility of Free-breathing CCTA using 256-MDCT

**DOI:** 10.1097/MD.0000000000004096

**Published:** 2016-07-08

**Authors:** Zhuo Liu, Ye Sun, Zhuolu Zhang, Lei Chen, Nan Hong

**Affiliations:** Department of Radiology, Peking University People's Hospital, Beijing, China.

**Keywords:** breath-holding, computed tomography, coronary CT angiography, free-breathing

## Abstract

Usually, coronary computed tomography angiography (CCTA) is performed during breath-holding to reduce artifact caused by respiration. The objective of this study was to evaluate the feasibility of free-breathing CCTA compared to breath-holding using CT scanner with wide detector. To evaluate the feasibility of CCTA during free-breathing using a 256-MDCT. In 80 patients who underwent CCTA, 40 were performed during breath-holding (group A), and the remaining 40 during free-breathing (group B). The quality scores for coronary arteries were analyzed and defined as: 3 (excellent), 2 (good), and 1 (poor). The image noise, signal-to-noise ratio and effective radiation dose as well as the heart rate variation were compared. The noise, signal-to-noise ratio, and effective radiation dose were not significantly different between the 2 groups. The mean heart rate variation between planning and scanning for group A was 7 ± 7.6 bpm, and larger than 3 ± 2.6 bpm for group B (*P* = 0.012). Quality scores of the free-breathing group were better than those of the breath-holding group (group A: 2.55 ± 0.64, group B: 2.85 ± 0.36, *P* = 0.018). Free-breathing CCTA is feasible on wide detector CT scanner to provide acceptable image quality with reduced heart rate variation and better images for certain patients.

## 1 Introduction

With continuous development of technologies, the diagnostic value of coronary computed tomography angiography (CCTA) is increasingly attached with importance in the clinical practice.^[1–4]^ CCTA examination usually requires the breath-holding cooperation of patients to reduce artifact caused by respiration. For patients who cannot hold breath, CCTA cannot be performed successfully. And sometimes, the heart rate variation caused from breath-holding may result in poor image quality or even failure. This study aimed to explore the feasibility of free-breathing CCTA using CT scanner with wide detector and high temporal resolution.

## 2 Materials and methods

### 2.1 General data

Eighty patients were randomly divided into 2 groups: group A (breath-holding, n = 40) and group B (free-breathing, n = 40). No heart rate control was performed before examination. Patients in group A received breathing training, while those in group B were ordered to breathe normally during examination. This study was approved by the ethics committee in our hospital, and written informed consent was acquired from each patient.

### 2.2 Scanning parameters

All examinations were performed using a 256-MDCT scanner (Revolution CT, GE Healthcare, Milwaukee, USA). The scanning range was from tracheal bifurcation to cardiac base for both groups. The maximal *z*-axis coverage range of detector was up to 160 mm. All data could be acquired using prospectively electrocardiogram-triggered axial scan during one tube rotation and within one R-R interval, without movement of the table. According to heart size, 120, 140, or 160 mm of *z*-axis coverage was chosen. The tube voltage was determined automatically by scanner based on scout images, and the options included 100 and 120 kVp. The tube current was also chosen automatically by scanner, ranging from 200 to 650 mA. The preset noise index was 25 HU. The slice thickness and interval were both 0.625 mm, and the matrix was 512 × 512. The gantry rotation speed was 0.28 s/rot. The standard reconstruction type was applied with hybrid iterative reconstruction algorithm (adaptive statistical iterative reconstruction-Veo, ASIR-V, GE Healthcare) at 60% blending percentage.^[5–7]^ A cardiac motion correction algorithm (snapshot freeze, SSF, GE Healthcare) was used during reconstruction to further increase temporal resolution.^[8,9]^

The scanner recorded electrocardiogram of up to 10 s before scanning, and selected optimal exposure phase according to the heart rate (Table 1).^[1,7]^ After scanning, images at the optimal phase were reconstructed. Axial images, volume rendering images and curved plannar reformation images were comprehensively evaluated.

**Table 1:**
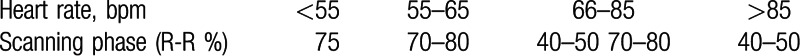
Automatic scanning phase selection by auto gating based on heart rates.

About 50 mL of the nonionic iodine contrast agent Iopromide (370 mgI/mL) was injected via antecubital vein at a flow rate of 5 mL/s followed by 20 mL of saline at the same rate. Automatic bolus tracking was applied to trigger the acquisition. The region of interest (ROI) was located in the descending aorta at the level of tracheal bifurcation, and the scan was started by a delay of 5.9 s after the CT value in ROI reached enhancement of 80 HU. The breath-holding instruction in group A took 5.9 s.^[10]^ Patients in group A were required to hold breath during scanning, while those in group B were required to breathe normally, without breath-holding instruction.

### 2.3 Evaluation on heart rate variation

The heart rates during planning and scanning were recorded and variations were calculated.

### 2.4 Evaluation on image quality

CT values of ROIs about 100 mm^2^ at the root of ascending aorta and standard deviations (SD) were measured for evaluating the image noise. Signal-to-noise ratios (SNR = CT value/SD), were also calculated.

**Figure 1:**
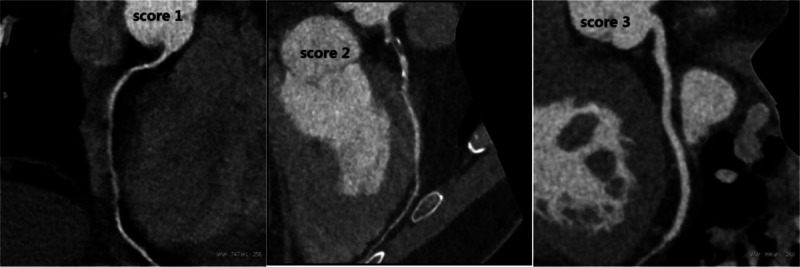
Subjective evaluation criteria (3: excellent; 2: good; and 1 poor).

Image quality of every coronary artery segment according to the American Heart Association 15-segment model with at least 1.0 mm diameter was evaluated using 3-point grading scales (3: excellent image quality without artifacts; 2: good image quality with minor artifacts; and 1: nondiagnostic image quality due to major artefacts).^[10,11]^ Two experienced radiologists, who were blinded to the fact whether the patient was breath-holder or not, evaluated the image quality of all datasets in consensus. The evaluation contents included the sharpness of inner and outer vascular walls, the degree of motion-related artifacts, and the border of plaque (calcified and noncalcified plaques) and lumen. The scores of all segments in a patient were averaged to give a final score. The scoring standard was shown in Fig. 1.

### 2.5 Radiation dose

The dose length product was recorded according to the dose report. The effective dose (ED) was calculated using a conversion coefficient for chest (k = 0.014 mSv/[mGy cm]).

### 2.6 Statistical analysis

Comparisons of age, body mass index (BMI), ED, heart rate variation, SD, and SNR between 2 groups were performed with independent *t*-test. The comparison of subjective evaluation scores was performed with Mann–Whitney *U* test. *P* < 0.05 suggested that a difference was statistically significant. The interobserver agreement was analyzed with Kappa test (κ < 0.40: poor agreement; 0.40 ≤κ <0.75: good agreement; and κ ≥ 0.75: excellent agreement). All statistical analyses were performed by (SPSS, Chicago, IL, USA) 20.0.

## 3 Results

### 3.1 General conditions

There were no statistically significant differences in BMI, age and ED between the 2 groups (*P* > 0.05). The BMI, age and ED of group A and group B were 25.15 ± 3.17 kg/m^2^, 57 ± 7 years, 1.88 ± 0.81 mSv and 26.56 ± 3.11 kg/m^2^, 59 ± 8 years, 1.91 ± 0.85 mSv, respectively.

### 3.2 Heart rate variation

The heart rate during scanning was 69 ± 10.8 bpm in group A and 70 ± 12.4 bpm in group B (*P* = 0.825). The heart rate during planning was 69 ± 11.3 bpm (50–114 bpm) in group A and 72 ± 12.2 bpm (52–102 bpm) in group B (p = 0.297). The variation between planning and scanning was 7 ± 7.6 bpm in group A and 3 ± 2.6 bpm in group B (*P* = 0.012; Table 2).

**Table 2:**
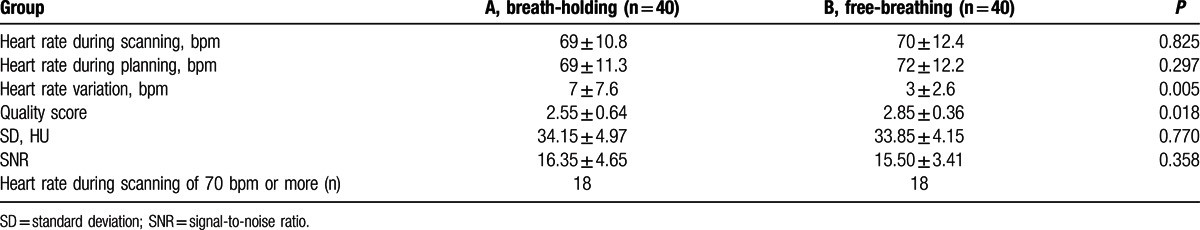
Comparison results between group A and group B (*P* < 0.05 indicated statistically significance).

### 3.3 Image quality

**Figure 2:**
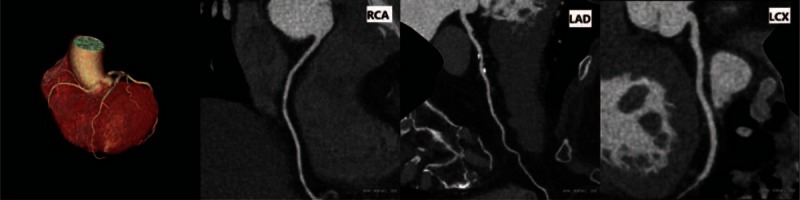
67-year-old female patient, free-breathing, heart rate during scanning: 52 bpm.

**Figure 3:**
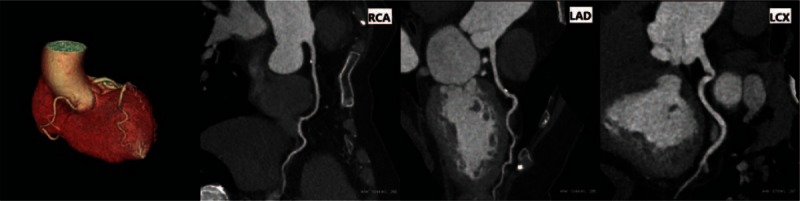
63-year-old female patient, free-breathing, heart rate during scanning: 102 bpm.

**Figure 4:**
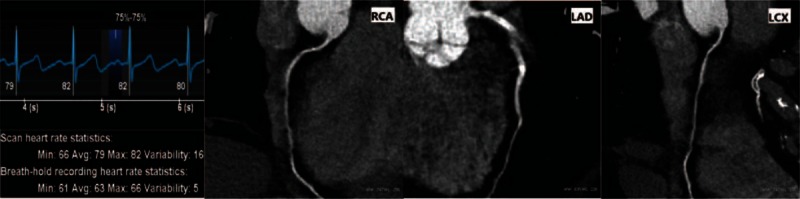
82-year-old female patient, breath-holding, mean heart rate during planning: 63 bpm, exposure phase 70%–80% R-R interval, heart rate during scanning: 82 bpm, quality score was 1.

There were no statistically significant differences in image noise and SNR between 2 groups (*P* > 0.05). The analysis of interobserver agreement in subjective evaluation score showed κ = 0.67, indicating good agreement. The subjective evaluation score in the free-breathing group was higher than that in the breath-holding group (Figs. 2–4, Table 2).

## 4 Discussion

In order to suppress the artifact caused by respiratory movement during scanning, patients are usually required to hold their breath during CCTA scanning. However, some patients cannot hold their breath or the heart rate increases during breath-holding. A significant variation in heart rate may occur, and result in poor image quality or even failure. The results of this study indicated that the heart rate variation between the planning and scanning in the breath-holding group was higher than the free-breathing group, and the free-breathing group had higher image quality score than breath-holding group.

We speculated the results above were due to the following reasons. First, a study showed that under the normal breathing condition (12–20 bpm), the movement speed of coronary artery caused by diaphragm movement was 6.4–29.3 mm/s.^[12]^ Another study demonstrated that the speed of coronary artery caused by heart beats was 22.4–108.6 mm/s.^[13]^ The former is much lower than the later, so the artifact caused by respiration could be neglected. Second, the development of CT scanner contributes to high temporal resolution, so that the motion artifact of coronary artery could be suppressed, including motion artifacts caused by the cardiac motion and respiratory motion. Third, compared with breath-holding, free-breathing CCTA reduced the heart rate variation caused by breath-holding instruction and thus increased the success rate in turn.

Previous literatures^[14,15]^ reported free-breathing CCTA by dual-source CT, and the heart rate was controlled under 60 bpm. Other scholars^[16,17]^ performed CCTA under free-breathing using 320-MDCT, and the heart rate was required to be 75 bpm or less due to the limitation of temporal resolution. It is thereby shown that free-breathing CCTA can be performed for patients with low heart rates, but there is no report about the feasibility for high heart rate (≥70 bpm). In this study, no heart rate control was performed. In the free-breathing group, 18 patients have heart rates of 70 bmp or more (maximal: 101 bpm), and the images were all acceptable for diagnosis (≥2).

The major limitation of this study is that no invasive angiography validation of results is performed, and image quality is subjective measure, since the aim of this study was to discuss whether free-breathing CCTA could reduce heart rate variation and motion artifact caused by respiration.

In summary, free-breathing CCTA is feasible using 256-MDCT scanner without heart rate control, and furthermore, can provide better image quality with reduced heart rate variation for certain patients.
